# Adaptation and validation of a food frequency questionnaire (FFQ) to assess dietary intake in Moroccan adults

**DOI:** 10.1186/s12937-018-0368-4

**Published:** 2018-06-12

**Authors:** Khaoula El Kinany, Vanessa Garcia-Larsen, Mohamed Khalis, Meimouna Mint Sidi Deoula, Abdelilah Benslimane, Amran Ibrahim, Mohamed Chakib Benjelloun, Karima El Rhazi

**Affiliations:** 1 Department of Epidemiology and Public Health, Faculty of Medicine and pharmacy of Fez, Sidi Mohamed Ben Abdellah University, Fez, Morocco; 2 Laboratory of Microbiology and Molecular Biology, Faculty of Science DharMehraz, Fez, Morocco; 3 Department of International Health, The Johns Hopkins Bloomberg School of Public Health, Baltimore, USA; 4 Population Health and Occupational Disease, NHLI, Imperial College London, London, UK; 5 Respiratory Department, Hassan II University Hospital Center of Fez, Fez, Morocco

**Keywords:** Food frequency questionnaire, Morocco, North Africa, Validity, Reproducibility, Diet, GA^2^LEN

## Abstract

**Background:**

To date, no culture-specific food frequency questionnaires (FFQ) are available in North Africa. The aim of this study was to adapt and examine the reproducibility and validity of an FFQ or use in the Moroccan population.

**Methods:**

The European Global Asthma and Allergy Network (GA^2^LEN) FFQ was used to assess its applicability in Morocco. The GA^2^LEN FFQ is comprised of 32 food sections and 200 food items. Using scientific published literature, as well as local resources, we identified and added foods that were representative of the Moroccan diet. Translation of the FFQ into Moroccan Arabic was carried out following the World Health Organization (WHO) standard operational procedure. To test the validity and the reproducibility of the FFQ, 105 healthy adults working at Hassan II University Hospital Center of Fez were invited to answer the adapted FFQ in two occasions, 1 month apart, and to complete three 24-h dietary recall questionnaires during this period. Pearson correlation, and Bland-Altman plots were used to assess validity of nutrient intakes. The reproducibility between nutrient intakes as reported from the first and second FFQ were calculated using intra-class correlation coefficient (ICC). All nutrients were log-transformed to improve normality and were adjusted using the residual method.

**Results:**

The adapted FFQ was comprised of 255 items that included traditional Moroccan foods. Eighty-seven adults (mean age 27.3 ± 5.7 years) completed all the questionnaires. For energy and nutrients, the intakes reported in the FFQ1 were higher than the mean intakes reported by the 24-h recall questionnaires. The Pearson correlation coefficients between the first FFQ and the mean of three 24-h recall questionnaires were statistically significant. For validity, de-attenuated correlations were all positive, statistically significant and ranging from 0.24 (fiber) to 0.93 (total MUFA). For reproducibility, the ICCs were statistically significant and ranged between 0.69 for fat and 0.84 for Vitamin A.

**Conclusion:**

This adapted FFQ is an acceptable tool to assess usual dietary intake in Moroccan adults. Given its representativeness of local food intake, it can be used as an instrument to investigate the role of diet on health and disease outcomes.

## Background

The burden of chronic non-communicable diseases (NCD) in African countries continues to rise [1]. The epidemiological profile of North Africa increasingly mirrors that of more developed societies, where cancer, cardiovascular, and respiratory diseases represent a major societal and health burden. Prevalence of these, and other NCDs related to diet, has continuously increased in the last two decades [2–4], but there is scant scientific evidence on the role of dietary habits on disease risk and prevalence in the Moroccan population [5, 6].

Food frequency questionnaires (FFQs) are a helpful instrument to ascertain usual dietary intake and its relationship with health and disease outcomes [7, 8]. Although FFQs are widely used in Europe and America [9, 10], nutritional epidemiology in Morocco remains hindered by the lack of locally representative dietary questionnaires, particularly FFQs. We are only aware of one FFQ recently developed to ascertain usual fruit and vegetable intake in Moroccan adults [11]. To date, the vast majority of what we know about dietary habits and chronic disease in this country relates to their association with Ramadan and obesity [2, 12].

The rapid socio-economic transition in North Africa has been accompanied by changes in the way the population eat, which are not easily captured with dietary questionnaires from, for example, high income countries. Morocco is a fast-growing developing country with a diet characterised by intake of vegetable-based dish, spices, and meat [11–13], and a rich combination of very traditional dishes with a more modern cuisine. Having FFQs that reflect such transitions and cultural features are urgently needed to identify regionally and locally relevant dietary risk factors for health and disease outcomes. To implement these FFQs, the validity and reproducibility of the instrument needs to be assessed [14, 15].

Our study was aimed at adapting the international GA^2^LEN FFQ to include staple foods consumed in Morocco, and at validating it in a sample of health Moroccan adults.

## Methods

### Participants

One hundred five adults working at Hassan II University Hospital Center of Fez were invited to answer the three 24-Hour Recall and the FFQ in two occasions. Eligibility to take part in the study was defined as having a regular diet over the previous 12 months and not have used any medications known to affect food intake or appetite during this period. The subjects had a stable weight. Data collection was conducted over a period of 4 months (July to October) in 2009.

### FFQ adaptation

The Global Asthma and Allergy Network (GA^2^LEN) FFQ was adapted to reflect the Moroccan diet. The GA^2^LEN FFQ was designed to be used as a single, common instrument to assess dietary intake across Europe [9]. It was initially piloted and validated in five European countries, and it has been subsequently used in several multi-national studies including high and low income countries [16].

To adapt the GA^2^LEN FFQ to the Moroccan diet we compiled information published in the scientific literature on usual foods commonly consumed in Morocco and these were added to each section. In order to retain its international comparability, several food items from the original GA^2^LEN FFQ were kept in each of the sections even though they were not necessarily relevant to the Moroccan diet (e.g. pork or alcohol intake).

The Standard Operational Procedure (SOP) of the World Health Organization [17] was followed for the forward and back translations from English to Moroccan Arabic. A first translation from English into Moroccan Arabic (*version 1*) was carried out by a bilingual person. This version was then tested amongst five people from the respiratory unit of the University Hospital of Fez. Doubts and difficulties in answering the questions were investigated and after this initial assessment, a second Arabic version was produced (*version 2*). To improve the identification of foods relevant to the Moroccan population, the research team in Fez also visited several local markets and supermarkets to identify common brand names and foods that could be relevant and were added accordingly, adding up to a total of 255 food items in the FFQ (Table 1). Subsequent back-translation into English was performed by another translator with a good knowledge of English but who had not seen the FFQ before. A final draft of the FFQ (*version 3*) was agreed in Moroccan Arabic and English (Table 1).

**Table 1:** Foods included in FFQ for Morocco

Name	
1-Bread	
Any type of bread	
Bread, whole meal, average (Durum Wheat)	
Bread, white, French stick	
Bread of zouane (Rye)	
Mllaoui/rghaif/mssemen/batbout/matlouaa	
Bread of smida/harcha (Semolina)	
Homemade bread	
Other type of bread (barley)	
2-Breakfast with grains	
Any type of grains	
Assida/Smida	
Dchicha/belboula	
Porridge (herbel), mflak	
All-Bran	
Corn Flakes	
3-Couscous	
Barley Couscous, cooked with meat, vegetables and dried grape	
Barley Couscous, cooked with sugar and cinnamon	
Wheat Couscous, cooked with meat, vegetables and dried grape	
Wheat Couscous, cooked with sugar and cinnamon	
Corn Couscous, cooked with meat, vegetables and dried grape	
Corn Couscous, cooked with sugar and cinnamon	
4-Pasta	
Any type of Pasta	
Pasta white boiled (Spaghetti, Macaroni)	
Pasta, whole meal, boiled	
Pasta with meat vegetables and cheese	
Chaaria Mhammsa	
5-Cake	
Any type of cake, cherry	
Madeleine cake	
Cake with date	
Croissants	
Moroccan swetees	
Basboussa Maqrout	
Aassida	
Doughnuts, ring	
Rice pudding, canned	
Pancake roll	
Cake, coconut	
Sellou Zammita	
Chabbakia Mkharrka	
6-Rice	
Any type of rice, brown, boiled	
Rice, white, easy cook, boiled	
Rice, brown, boiled	
Noodles, rice, dried	
7-Sugar	
Sugar, white	
Jam, fruit spread	
Honey	
Syrup, golden	
8-Sweets without chocolate	
Chew sweets	
Fudge	
Toffees	
Cereal chewy bar	
Polo skimo glace	
9-Chocolate	
Any type of chocolate	
Chocolate covered bar with fruit/nut/bix	
Natural white and black chocolate	
10-Vegetable oil	
Oil, vegetable, blended, average	
Oil, safflower	
Oil, olive	
Oil, Argan	
Oil, corn	
11-Margarine and vegetable fat	
Any margarine and vegetable fat, (except soya fat)	
Light margarine or less fat (30% fat)	
Margarine (from 40 to 60% fat)	
Normal margarine (more than 70% fat)	
Mixed fat (except soya)	
Original fat of soya (any type)	
12-Butter and animals fat	
Any animal fat (butter)	
Butter with less fat (40% less fat)	
Butter with less fat (from 40 to 60% fat)	
Smen (traditional butter)	
13-Nuts	
Any type of dried Fruit	
Peanuts, plain	
Cashew nuts, roasted & salted	
Almonds toasted	
Walnuts	
Pistachio nuts, roasted & salted	
Chestnuts	
Oak nut	
14-Legumes	
Any legumes	
white beans, boiled	
Lentils, red, split, boiled	
Chick peas, whole, dried, boiled unsal	
Green beans/French beans, raw	
Broad beans, frozen, boiled in unsalted	
Soya beans, dried, boiled	
Peas, raw	
15-Vegetables (mean dish)	
Any vegetables except potatoes	
Lettuce, average, raw	
Spinach, raw	
Fenugreek seeds	
Rejla; Bakkoula	
Mloukhia (jews Mallow)	
Tomatoes, raw	
Aubergine, raw (Eggplant)	
Courgette, raw (squash)	
Peppers, red, raw, yellow	
Cucumber, raw	
Carrots, raw	
Parsnip, raw	
Swede, raw	
Artichoke globe, raw	
Radish, white, mooli, raw	
Beetroot, raw	
Chilli peppers, green, raw	
Sweet corn Kernels, raw	
Asparagus, raw	
Aromatic herbs (Mint basilica, parsley basil coriander)	
Leeks, raw	
Mushrooms, black, white	
Onions, raw	
Garlic, raw	
Cauliflower, raw	
Pumpkin red	
Brussels sprouts, raw	
Broccoli, green, raw	
Cabbage white, red, green	
Tomatoes stuffed with vegetables	
Pickle, mixed veg	
Ginger, root	
16-Potatoes (mean dish)	
Any type of potatoes	
Potatoes, old mashed with hard marg	
Potatoes, old, baked, flesh & skin	
Chips, homemade, fried in blended oil	
Salad, potato with French dressing	
Potato cakes fried in veg oil	
Tortillas	
Sweet potato	
17-Fruits (one unit)	
Any type of fruits	
Apples	
Pears	
Bananas	
Peaches	
Avocado	
Cherries	
Lemon pickles	
Mulberries, raw, Blackcurrants, Raspberries	
Watermelon	
Grapes	
Mangoes	
Apricots	
Nectarines	
Plums	
Dried mixed fruit	
Pineapple	
Kiwi Fruit	
Juice, lemon	
Oranges	
Mandarine	
Grapefruit	
Fruit cocktail, conserved in syrup	
Figs, raw, dried	
Black or green olives	
Raisins	
Dates, dried with stones	
18-Juice	
Orange juice (concentrate)	
Pomegranate juice (pomegranate, raw)	
Any other type of juice	
19-Non-alcoholic beverages	
Lemonade	
Beet juice	
Mineral water	
20-Coffee/Tea	
Tea, infusion	
Coffee, instant, made up	
Zizwa (coffee, liquid)	
Tea, Chinese, leaves, infusion	
Mint, fresh	
Other herbal infusions	
21-Beer	
Any type of beer	
22-Wine	
Any type of wine	
Wine, red	
Wine, white, dry	
Wine, rose	
23- Other-alcoholic beverages	
Port, sherry, liqueur,	
Spirits 37.5%	
24-Red meat	
Any type of red meat (beef, cow, lamb, goat)	
Beef, fillet steak, forerib, lean & fat, roast, steamed, grilled	
Beef in tagine	
Minced meat of beef	
Lamb, grilled, steamed, roasted	
Lamb cooked in tagine, Mrouzia	
Minced meat of lamb	
Goat meat	
Veal, fillet, roast	
Camel meat	
Rabbit, Duck, partridge	
Sausage of beef, lamb, cow, chilled, fried	
kocha or bread filled with meat	
Kabab, chawarma	
Pork	
Khliaa/Dried meat	
Khliaa (dried meat with salt and cooked with fat), cow	
Khliaa (dried meat with salt and cooked with fat), sheep	
Qaddid (dried meat with salt), cow, sheep	
Dried pork meat	
25-Poultry	
Any type of chicken	
Chicken steamed	
Chicken cooked in tagine	
Chicken grilled and roasted	
Turkey steamed	
Turkey cooked in tagine	
Turkey grilled and roasted	
Sausage and skewer of turkey	
Poultry smoked, conserved	
Any poultry smoked, conserved (e.g. mortadella, casheer)	
26-Offal (sekat)	
Liver of beef, lamb	
Tongue, heart, kidney, head, brain, of cow or beef or sheep, lamb	
27-Fish	
Any fish fresh, smoked, white, fat	
Fresh fat fish (e.g. salamon, tuna, truite, sardine, bouri)	
White fresh fish (e.g. sole, merlan)	
Fresh fish / other sea foods (eggs of fish)	
Seafood shrimp, squid, mussels	
Frozen seafood	
Frozen fat fish (e.g. salamon, tuna, truite, sardine, bouri)	
Frozen white fish (e.g. sole, merlan)	
Conserved fat fish (e.g. salamon, tuna, truite, sardine, bouri)	
Fat fish dried or smoked (e.g. salamon, tuna, truite, sardine, bouri)	
White fish dried or smoked (e.g. sole, merlan)	
Conserved seafood shrimp, squid, mussels	
28-Eggs	
Farmer eggs	
Farmer egg boiled or sandwich	
Farmer eggs’s meals: Omlet, eggs with tomatos, eggs with pepper and tomatos	
Dessert with Farmer eggs (Cake, egg tart)	
Industrial eggs	
Industrial egg boiled or sandwich	
Industrial egg’s meals: Omlet, eggs with tomatos, eggs with pepper and tomatos	
Dessert with Industrial eggs (Cake, egg tart)	
29-Milk of cow/Milk of soya	
Whole milk (milk,cow,whole,3,5%fat)	
Lben (alone or with fruit)	
Skimmed milk (Milk, cow, skimmed, 0,5% fat)	
Semi skimmed milk (Milk, cow, partly skimmed, 1.5% fat)	
Milk free fat	
Raib	
Soya milk	
Saykook	
Yaourt	
Yaourt Activia	
Soya yaourt	
30-Cheese	
Any type of cheese	
Hard cheese (e.g. Cheddar, Parmesan)	
Soft cheese (Camembert, Brie, Philadelphia)	
Semi hard Cheese (Gouda, Emmental/Edam)	
Jben (Natural or aromatic)	
Fresh cheese (e.g. Vita, Mozarelle)	
Others: La vache qui rit, Kiri, Coeur du lait, Junior	
31-Other dairy products	
Ice cream	
Cream	
Fresh cream	
Double cream	
32- Miscellaneous foods	
Soup with vegetables and meat	
Soup with vegetables and grains (e.g. Dchicha, Smida)	
Soup with meat or offal	
Soup with fish	
Tagine with meat or poultry	
Salt brik	
Pizza	
Sorghum	
Chilli sauce	
Ketchup	
Salad sauce	
Mayonnaise	
Mustard	

Each food item in the FFQ was assigned a portion size using standard local household units such as plate, bowl, spoons of different size (tablespoon, teaspoon), tea-pot, tea-glass, and glass of water, as well as using photographs from a booklet (‘Food and typical preparations of the Moroccan population’ [14].

Frequency of dietary intake reported in the FFQ was estimated by selecting one of eight categories: never, once to three times per month, once a week, twice to four per week, five to six times per week, once per day, twice to three times, more than four times.

### Validation of the FFQ

**Fig. 1:**
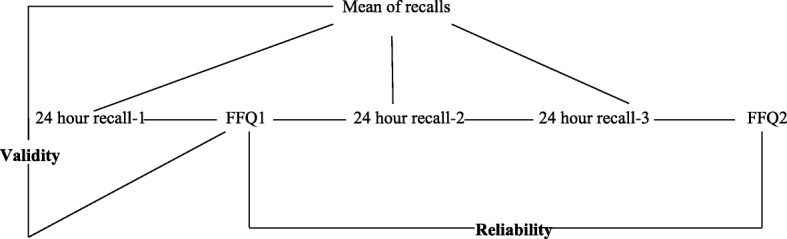
Schematic representation of the study aiming to test the relative validity and reliability of the Moroccan Food Frequency Questionnaire against 24-hour recalls

The FFQ was validated against the average of three 24-h recall questionnaires over a period of 1 month (Fig. 1). Participants were first asked to answer a 24-h recall questionnaire, where they reported all the foods and beverages consumed the day before, providing qualitative (e.g. type of food) and quantitative (e.g. portions) details. Each of the three 24-h recall questionnaires was administered 10 days apart, on two working days and 1 week-end day. The recalled food items were assigned to the food groups of the adapted FFQ.

The FFQ was completed in two occasions, a month apart, a day after participants completed the first and last 24-h recall questionnaires.

### Nutritional composition data for Moroccan foods

Available Food Composition Tables from Morocco were used to derive nutrient composition for several traditional dishes and for some modern products [14, 15]. Additional information needed for non-traditional (‘modern’) foods was obtained from other regional sources of data, namely the Tunisian food composition table [18], the food composition table for African countries (FAO) [19], the French food composition table (CIQUAL) [20] and the United States department of agriculture nutrient database (USDA) [21].

To calculate total energy intake (TEI), macro-, and micro-nutrient intakes, we created a syntax using the SPSS.20 software. First, the amount of servings consumed was estimated using the standard food portion sizes and these were converted into grams per day [14]. For seasonal foods, participants were asked to answer the question based on intake when these foods were available. The daily intake was calculated according to the number of months per year that each seasonal food was available. TEI and nutrient intakes were calculated by multiplying the frequency of consumption of each food item by the content (per 100 g) and by the specified portion, and then adding the contribution from all food items.

### Socio-demographic characteristics

The FFQ had an additional section enquiring about general characteristics namely age, sex, educational level, and occupation. To estimate body mass index (BMI), height and weight were measured using a calibrated equipment (stadiometer and weighing scale, respectively) and BMI was derived using the formula weight (kg) divided by height^2^ (m^2^).

### Statistical analyses

Descriptive results were expressed as means standard deviations, or as percentages and frequencies for continuous and qualitative variables, respectively.

The mean daily intake of the three 24-h recall questionnaires was used as a representative average of the consumption reported in these questionnaires. Descriptive means and standard deviations of nutrient intakes estimated by the FFQ the first and second time (FFQ1 and, FFQ2), and the average of the three 24-h recall questionnaires are presented as untransformed values. As nutrient variables were not normally distributed these were log-transformed (log10) to reduce skewness and optimize the normality of the distribution.

Validity of the FFQ1 was compared with the average of three 24-h recall questionnaires using Pearson correlation coefficients. Adjustment correlation coefficients for TEI were calculated using the residual method [22] (with TEI as the independent variable and the nutrient as the dependent variable). Energy adjusted intakes were calculated by adding the mean nutrient intake to the residual derived from the regression analysis. The de-attenuated correlations [23] were calculated to remove the within-person variability found in the 24-h recall questionnaires using the following formula:

$$ {\mathrm{r}}_t={\mathrm{r}}_0\sqrt{1+r/n} $$

**r**_**t**_ is the corrected correlation between the energy adjusted nutrient derived from the FFQ and 24-h recall questionnaires, **r**_**0**_ is the observed correlation, **r** is the ratio of estimated within-person and between- person variation in nutrient intake derived from the three 24-h recall questionnaires, and **n** is the number of replicated recalls (*n* = 3).

Bland–Altman plots [24, 25] were used to assess agreement between the two methods. For this analysis, the average values of FFQ1 and three 24 Hour Recalls ((FFQ1 + Mean 24 HRs)/2) were plotted against the difference in intake between the two methods, and the limits of agreements (mean difference ± 1.96 SD (differences)) were used to show how large the disagreements between the two methods.

For the reproducibility of the FFQ, the agreement between FFQ1 and FFQ2 was assessed by Pearson product-moment correlation coefficients and intra-class correlation coefficients (ICC) of transformed nutrients and energy-adjusted nutrient intakes. Statistical analyses were performed using SPSS 20.0.

### Participant’s consent and ethics

All participants were informed about their role in the study and gave formal consent before being interviewed. The study was approved by the Ethics Committee at University of Fez.

## Results

The final version of the adapted FFQ contained 255 foods, which were classified into 32 groups as follows: (1) bread, (2) breakfast with grains, (3) couscous, (4) pasta, (5) cake, (6) rice, (7) sugar, (8) sweets without chocolate, (9) chocolate, (10) vegetable oil, (11) margarine and vegetable fat, (12) butter and animals fat, (13) dried fruit, (14) legumes, (15) vegetables, (16) potatoes, (17) fruits, (18) juice, (19) non-alcoholic beverages, (20) coffee/tea, (21) beer, (22) wine, (23) other-alcoholic beverages, (24) red meat, (25) poultry, (26) sekat (offal), (27) fish, (28) eggs, (29) milk of cow/milk of soya, (30) cheese, (31) other dairy products, and (32) miscellaneous foods (Table 1).

A total of 87 participants completed all the dietary questionnaires (two FFQs and three24-h recall questionnaires). Most of the participants were females (70.1%) and young adults (mean age 27.3 ± 5.7 years). Over two thirds of participants (70.6%) had a normal BMI (Table 2). Eighteen subjects did not complete the second FFQ, with the main reason being declining to participate again (*n* = 12), or not being available after several attempts were made to contact them (*n* = 6).

**Table 2:** Socio-demographic characteristics and anthropometric measurements of study participants (*N* = 87)

Characteristics	Results
Age (mean ± SD)	27.3 ± 5.6
Gender (%)	
Female	70.1
Male	29.9
Education (%)	
Primary	2.3
Secondary	10.3
University	87.4
Body masse index category (%)	
Underweight (< 18.5)	3.5
Normal (18.5–24.9)	70.6
Overweight (25–29.9)	22.4
Obese (BMI ≥30)	3.5

**Fig. 2:**
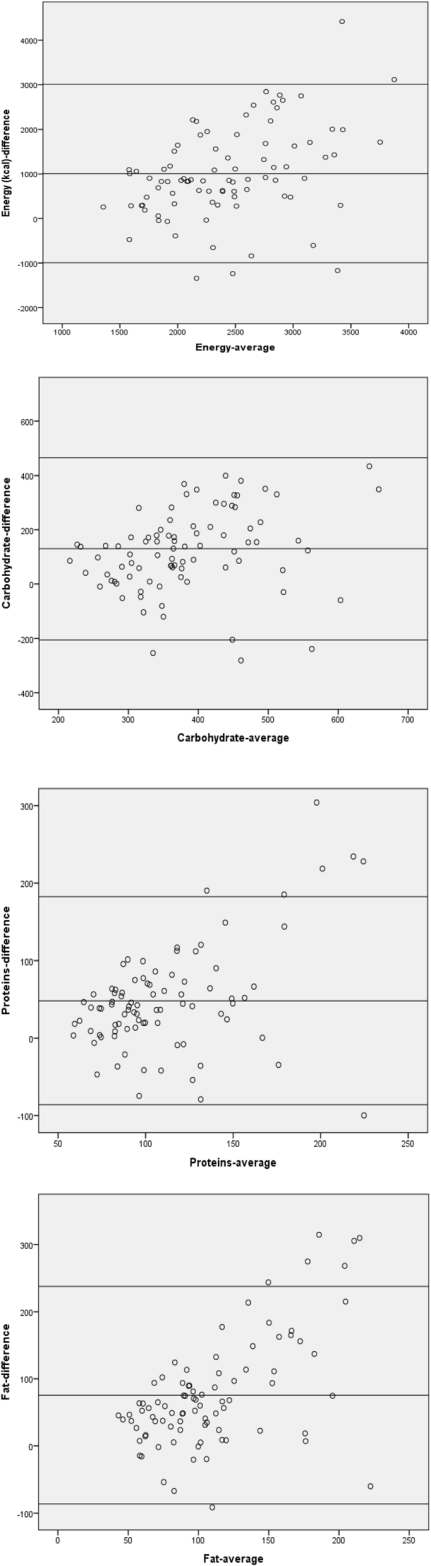
Bland altman plots of difference between energy and macro-nutrients (carbohydrate, proteins, and fat) as predicted by the first FFQ and the mean of three 24-hour recalls

The mean intake of TEI, macro-nutrients and micro-nutrients measured by FFQ1, FFQ2, and the 24-h recall questionnaires are presented in Tables 3. For TEI and nutrients intakes, the means reported in the FFQ1 were higher than the means reported using the average of the three 24-h recall questionnaires. The Bland-Altman plots for energy, and macronutrients (carbohydrates, proteins, and fat) are shown in Fig. 2. The Bland Altman plots confirmed an over-estimation of nutrient intakes consumptions by the FFQ.

**Table 3:** Daily consumption of nutrients estimated by the first and second Food Frequency Questionnaire and mean of three 24 Hour Recalls

Nutrients	FFQ1	FFQ2	24 Hour Recalls
	Mean ± SD	Mean ± SD	Mean ± SD
Energy (kcal)	2546.5 ± 719.5	2392.5 ± 738.9	1926.2 ± 589.6
Carbohydrates(g)	452.1 ± 149.7	430.4 ± 148.6	321.9 ± 103.3
Proteins (g)	135.3 ± 61.6	128.9 ± 57.4	87.1 ± 38.2
Fat (g)	108.2 ± 39.9	103.9 ± 44.3	71.8 ± 39.0
Total MUFA(g)	110.2 ± 64.0	104.6 ± 57.8	45.8 ± 32.9
Total PUFA(g)	78.3 ± 53.8	72.5 ± 47.1	31.1 ± 29.9
Total SFA(g)	80.9 ± 55.6	75.6 ± 54.3	41.4 ± 33.1
Vitamin A (μg)	445.1 ± 220.9	439.9 ± 259.2	533.1 ± 680.8
Vitamin C (mg)	221.6 ± 141.6	196.3 ± 114.0	129.3 ± 93.4
Vitamin E (mg)	73.4 ± 53.1	70.1 ± 48.7	28.0 ± 30.2
Selenium (μg)	138.4 ± 74.0	144.7 ± 67.2	91.1 ± 63.7
Magnesium (mg)	567.3 ± 237.0	556.7 ± 230.3	324.7 ± 143.7
Calcium (mg)	1241.6 ± 600.6	1188.7 ± 576.2	755.0 ± 408.2
Iron (mg)	28.5 ± 22.4	26.7 ± 20.6	16.9 ± 11.4
Fiber (g)	49.4 ± 58.5	44.9 ± 52.1	26.3 ± 37.4

Correlations between nutrient intakes derived from the FFQ1 and the mean of the 24-hour recall questionnaires are presented in Table 4. Crude correlation coefficients between the two methods varied from 0.23 (fiber) to 0.89 (total monounsaturated fatty acids [MUFA]), and were statistically significant. Adjusting for TEI was statistically significant for all nutrients but it decreased the value of correlation coefficients. However, de-attenuation (adjustment for residual measurement error) increased all correlation coefficients, ranging from 0.24 (fiber) to 0.93 (total MUFA).

**Table 4:** Validity of Food Frequency Questionnaire: Pearson correlations between first food frequency questionnaire and mean of three 24 Hour Recalls

Nutrients	24 Hour Recalls Vs Food Frequency Questionnaire1		
	Unadjusted	Energy adjusted	De-attenuated
Energy (kcal)	0.67^*^	–	0.69^*^
Carbohydrates(g)	0.63^*^	0.60^*^	0.66^*^
Proteins (g)	0.34^*^	0.29^*^	0.35^*^
Fat (g)	0.26^*^	0.19^*^	0.28^*^
Total MUFA(g)	0.89^*^	0.86^*^	0.93^*^
Total PUFA(g)	0.87^*^	0.84^*^	0.91^*^
Total SFA(g)	0.79^*^	0.82^*^	0.90^*^
Vitamin A (μg)	0.55^*^	0.52^*^	0.72^*^
Vitamin C (mg)	0.62^*^	0.40^*^	0.63^*^
Vitamin E (mg)	0.71^*^	0.70^*^	0.74^*^
Selenium (μg)	0.36^*^	0.33^*^	0.38^*^
Magnesium (mg)	0.56^*^	0.43^*^	0.66^*^
Calcium (mg)	0.46^*^	0.42^*^	0.55^*^
Iron (mg)	0.69^*^	0.58^*^	0.74^*^
Fiber (g)	0.23^*^	0.21^*^	0.24^*^

^*^Energy and nutrients were transformed (log10) to improve normality ^*^p ≤ 0.01

The intra-class correlation coefficients (ICC) and Pearson’s correlation coefficients for both the unadjusted and the energy adjusted nutrient intakes estimated from FFQ1 and FFQ2 were presented in Table 5. The Pearson correlations (unadjusted) between nutrient intakes assessed by two FFQ varied from 0.62 (carbohydrates) to 0.76 (Vitamin A). Adjusting for total energy intake decreased all correlation coefficients, ranging from 0.53 (fat) to 0.73 (Vitamin A). The ICCs unadjusted ranged from 0.76 (carbohydrates) to 0.86 (Vitamin A and Vitamin C). The ICCs energy adjusted ranged from 0.69 (fat) to 0.84 (Vitamin A). All correlations were statistically significant.

**Table 5:** FFQ reproducibility: Pearson correlation coefficients and intra-class correlation coefficients (ICC) for nutrient intake as reported in FFQ_t1_ and FFQ_t2_ in Moroccan adults

Nutrients	Pearson correlation coefficient		Intra-class correlation coefficient	
	Unadjusted	Energy-adjusted	Unadjusted	Energy-adjusted
Energy (kcal)	0.73^**^	–	0.84^**^	–
Carbohydrates(g)	0.62^**^	0.56^**^	0.76^**^	0.72^**^
Proteins (g)	0.68^**^	0.60^**^	0.81^**^	0.75^**^
Fat (g)	0.69^**^	0.53^**^	0.81^**^	0.69^**^
Total MUFA(g)	0.71^**^	0.61^**^	0.82^**^	0.76^**^
Total PUFA(g)	0.70^**^	0.61^**^	0.83^*^	0.76^**^
Total SFA(g)	0.73^*^	0.64^**^	0.84^**^	0.78^**^
Vitamin A (μg)	0.76^**^	0.73^**^	0.86^**^	0.84^**^
Vitamin C (mg)	0.75^**^	0.67^**^	0.86^**^	0.80^**^
Vitamin E (mg)	0.71^**^	0.60^**^	0.83^**^	0.75^**^
Selenium (μg)	0.66^**^	0.60^**^	0.80^**^	0.75^**^
Magnesium (mg)	0.64^**^	0.59^**^	0.78^**^	0.74^**^
Calcium (mg)	0.69^**^	0.64^**^	0.81^**^	0.78^**^
Iron (mg)	0.71^**^	0.66^**^	0.83^**^	0.80^**^
Fiber (g)	0.72^**^	0.65^**^	0.84^**^	0.79^**^

^*^Energy and nutrients were transformed (log10) to improve normality; ^**^p ≤ 0.001

## Discussion

Our study described the process of adaptation of the international GA^2^LEN FFQ for use in Moroccan adults, and its relative validity and reproducibility to estimate usual food intake. The adapted FFQ contained 255 items, including staple foods consumed by the Moroccan population. The FFQ was classified into 32 food groups or sections, to mirror the structure of the GA^2^LEN FFQ, which facilitates international comparability. To our knowledge, this is the first FFQ in Morocco to include a comprehensive list of both traditional and ‘modern’ foods, providing a reasonable assessment of relative dietary intake over a 1-year period. We are aware of another FFQ developed in Morocco by Landais et al., but it is limited to intake of fruits and vegetables only [11]. The energy adjusted Pearson correlation between the FFQ and the mean 24-HRs showed that the relative validity findings were moderately consistent across the majority of nutrients, they ranged between 0.19 for fat to 0.86 for total MUFA, and these observed values were comparable to other FFQs validation studies [26–28].

The nutrient intakes reported with the use of the FFQ were higher than those reported using the 24-h recall questionnaires. This over-reporting is not uncommon when validating an FFQ with a relatively large number of food items [26, 29–33]. We used the average of three 24-h recall questionnaires, which is considered an acceptable number of days to capture usual intake [34]. A systematic review found that 75% of validation studies use the 24-h recall questionnaires as reference method against FFQs [35], preferred for the accuracy to capture daily consumption of a varied diet, and for their relatively easier administration and analysis compared to other dietary questionnaires. The FFQ and the 24-h recall questionnaire have some differences in their error sources, which make them sufficiently independent [36]. Both instruments are prone to memory bias (long-term vs short term in the FFQ vs the 24-h recall questionnaire, respectively) and have differences in the perception of portion sizes (usually pre-defined in the FFQ) [35, 37, 38]. The 24-h recall questionnaire method is based on open-ended questions; while the FFQ is usually designed to have close-ended questions.

The acceptable correlations between the the FFQs and 24-HRs and the overestimation of energy and nutrient intakes between the two methods were confirmed by the Bland-Altman plots. These figures indicated a positive mean difference for TEI and macronutrients. These results are in agreement with those reported by other studies [39–41].

Since no dietary method can assess nutrient intake without error [35], we used energy adjusted nutrient estimates in our analyses as a way to reduce correlated errors between the two dietary methods [22, 38]. Energy-adjustment decreased correlation coefficients for all nutrients, which often happens when variability is more related to systematic errors of under/overestimation than to energy intake [42–44]. Similarly, other studies have not reported that energy-adjusted estimates improved crude correlations [45–47]. The de-attenuated correlations were increased because of the correction for the day to day variation in intakes.

The reproducibility of the FFQ was examined by the administration of the questionnaire in two occasions, 1 month apart. As reported in other studies [48, 49], we found that the estimates observed in FFQ1 were slightly higher than in the second FFQ. This could be partly explained by the level of engagement of the participants and the attention required to complete the FFQ in full. The ICCs showed a good level of agreement for the reporting of macro- and micronutrients, ranging from 0.69 (fat) to 0.75 (proteins for macro-nutrients, and over 0.7 for most micro-nutrients, suggesting that the FFQ has a good repeatability and reproducibility [50].

Our study has several strengths. The structure of the FFQ was adapted from the international GA^2^LEN FFQ, whose applicability has been demonstrated in multinational studies in high [9] and low income countries [51]. In order to make the FFQ representative of the Moroccan population, we endeavored to identify traditional foods that are part of the staple diet of the country, while also maintaining the international structure of the food classification to facilitate international comparisons. We followed a strict protocol to ensure the FFQ was correctly translated into Moroccan Arabic, which is different from the written and spoken Arabic in other North African countries. The FFQ also takes into account seasonal variations in food consumption, an important feature in North Africa where seasonality strongly influences dietary choices.

We acknowledge this validation study has some limitations. The FFQ captures usual intake of foods over a longer period of time than a 24-h recall questionnaire, which could lead to errors in the results. We compared the FFQ to the average intake reported in three 24-h recall questionnaires. Although this is an acceptable number of interviews, several studies recommend seven recall days (replicates) to capture a better estimate of the habitual intake. However, three recording days per subject are considered feasible and sufficient to estimate within-person variability (day-to-day variability). Due to the length of the validation study (1 month), some seasonal variations might not have been captured accurately with the 24-h recall questionnaire. This may negatively impact the correlation results, reflecting differences between the two instruments, rather than limitations of the FFQ. The length of the FFQ (255 food items) might have discouraged the participants to respond it fully. We designed the FFQ bearing in mind the current gap in nutritional epidemiology in North Africa, creating a tool that captures the usual diet of Morocco, and that it estimates intake of other foods that are associated with the nutritional transition of the region. Finally, the majority of the study sample was comprised of women with a high level of education. This does not represent the general population of Morocco, where illiteracy and poverty are common. The use of the FFQ in the general population would probably require a close interaction between an interviewer and the participant to overcome communication and educational limitations.

## Conclusions

This adaptation and validation study showed that the FFQ has a good relative validity and a good reproducibility for most nutrients. It is the first complete and validated tool to assess usual dietary intake in the Moroccan population that includes a wide range of traditional, as well as more ‘modern’ food items. Given its representativeness of local foods and habits, it can be used as an instrument to assess the relation of dietary habits and diseases in which diet might play a role.

## Abbreviations

BMI: Body mass index
EPIC: European Prospective Investigation into Cancer and Nutrition
FAO: Food and Agriculture Organization
FCT: Food composition table
FFQ: Food frequency questionnaire
GA^2^LEN: The European Global Asthma and Allergy Network
ICC: Intra-class correlation coefficient
MUFA: Monounsaturated fatty acids
NCD: Non-communicable diseases
PUFA: Polyunsaturated fatty acids
SFA: Saturated fatty acids
SOP: The standard operational procedure
WHO: World Health Organization
